# Treatment Patterns and Attrition With Lines of Therapy for Advanced Urothelial Carcinoma in the US

**DOI:** 10.1001/jamanetworkopen.2024.9417

**Published:** 2024-05-02

**Authors:** Vinay Mathew Thomas, Yeonjung Jo, Nishita Tripathi, Soumyajit Roy, Beverly Chigarira, Arshit Narang, Georges Gebrael, Chadi Hage Chehade, Nicolas Sayegh, Gliceida Galarza Fortuna, Richard Ji, Patrick Campbell, Haoran Li, Neeraj Agarwal, Sumati Gupta, Umang Swami

**Affiliations:** 1 Division of Medical Oncology, Department of Internal Medicine, Huntsman Cancer Institute, University of Utah, Salt Lake City; 2 Department of Internal Medicine, Detroit Medical Center Sinai Grace Hospital, Detroit, Michigan; 3 Department of Radiation Oncology, Rush Cancer Center, Chicago, Illinois; 4 Department of Internal Medicine, University of Texas Southwestern Medical Center, Dallas; 5 Division of Medical Oncology, Department of Internal Medicine, University of Kansas Cancer Center, Westwood

## Abstract

**Importance:**

The treatment paradigm for advanced urothelial carcinoma (aUC) has undergone substantial transformation due to the introduction of effective, novel therapeutic agents. However, outcomes remain poor, and little is known about current treatment approaches and attrition rates for patients with aUC.

**Objectives:**

To delineate evolving treatment patterns and attrition rates in patients with aUC using a US-based patient-level sample.

**Design, Setting, and Participants:**

This retrospective cohort study used patient-level data from the nationwide deidentified electronic health record database Flatiron Health, originating from approximately 280 oncology clinics across the US. Patients included in the analysis received treatment for metastatic or local aUC at a participating site from January 1, 2011, to January 31, 2023. Patients receiving treatment for 2 or more different types of cancer or participating in clinical trials were excluded from the analysis.

**Main Outcomes and Measures:**

Frequencies and percentages were used to summarize the (1) treatment received in each line (cisplatin-based regimens, carboplatin-based regimens, programmed cell death 1 and/or programmed cell death ligand 1 [PD-1/PD-L1] inhibitors, single-agent nonplatinum chemotherapy, enfortumab vedotin, erdafitinib, sacituzumab govitecan, or others) and (2) attrition of patients with each line of therapy, defined as the percentage of patients not progressing to the next line.

**Results:**

Of the 12 157 patients within the dataset, 7260 met the eligibility criteria and were included in the analysis (5364 [73.9%] men; median age at the start of first-line treatment, 73 [IQR, 66-80] years). All patients commenced first-line treatment; of these, only 2714 (37.4%) progressed to receive second-line treatment, and 857 (11.8%) advanced to third-line treatment. The primary regimens used as first-line treatment contained carboplatin (2241 [30.9%]), followed by PD-1/PD-L1 inhibitors (2174 [29.9%]). The PD-1/PD-L1 inhibitors emerged as the predominant choice in the second- and third-line (1412 of 2714 [52.0%] and 258 of 857 [30.1%], respectively) treatments. From 2019 onward, novel therapeutic agents were increasingly used in second- and third-line treatments, including enfortumab vedotin (219 of 2714 [8.1%] and 159 of 857 [18.6%], respectively), erdafitinib (39 of 2714 [1.4%] and 28 of 857 [3.3%], respectively), and sacituzumab govitecan (14 of 2714 [0.5%] and 34 of 857 [4.0%], respectively).

**Conclusions and Relevance:**

The findings of this cohort study suggest that approximately two-thirds of patients with aUC did not receive second-line treatment. Most first-line treatments do not include cisplatin-based regimens and instead incorporate carboplatin- or PD-1/PD-L1 inhibitor–based therapies. These data warrant the provision of more effective and tolerable first-line treatments for patients with aUC.

## Key Points

**Question:**

What are the current treatment patterns and attrition rates in patients with advanced urothelial cancer?

**Findings:**

In this cohort study of 7260 patients with advanced urothelial cancer who received first-line treatment, only 2714 (37%) progressed to second-line treatment, and 857 (12%) reached third-line treatment. The most common first-line regimens were carboplatin and programmed cell death 1 and/or programmed cell death ligand 1 inhibitors; novel therapeutic agents like enfortumab vedotin, sacituzumab govitecan, and erdafitinib have increased adoption after 2019.

**Meaning:**

The attrition rates observed in this study emphasize the necessity for more effective and tolerable front-line treatment options for patients with advanced urothelial cancer.

## Teaser

This cohort study assesses treatment patterns and attrition rates in patients with advanced urothelial carcinoma in US oncology clinics.

## Introduction

An estimated 82 290 new cases and 16 710 deaths are projected to be attributable to bladder cancer in the US in 2023.^1^ Although the 5-year survival rate for localized bladder cancer is 71%, the rate drops significantly to 8.3% for metastatic bladder cancer.^2^ Urothelial (transitional cell) carcinomas are the most common histological subtype of bladder cancer.^3^ The treatment landscape of urothelial cancer has evolved significantly over the past few years. Cisplatin-based chemotherapy currently remains a standard of care for metastatic and locally advanced urothelial cancer (hereafter referred to as aUC) in the first-line setting.^4,5,6^ Carboplatin with gemcitabine remains a viable front-line treatment choice for patients with aUC deemed ineligible for cisplatin-based therapy.^7^ Several programmed cell death 1 and/or programmed cell death ligand 1 (PD-1/PD-L1) inhibitors are approved for use in aUC. Pembrolizumab, nivolumab, and avelumab are approved for patients with aUC who have experienced disease progression during or following platinum-based chemotherapy.^8,9,10,11^ Pembrolizumab is also approved for use in the first-line setting for patients with aUC ineligible for any platinum-containing chemotherapy.^8,12^ Avelumab is approved for maintenance therapy for patients with aUC whose disease has not progressed following initial platinum-based treatment.^8,13^

The pan–fibroblast growth factor receptor (FGFR) inhibitor erdafitinib has been granted accelerated approval for patients with aUC with susceptible FGFR2 or FGFR3 genetic alterations and whose disease has progressed during or following platinum-based therapy.^8,14^ Enfortumab vedotin is a nectin-4–directed antibody and microtubule inhibitor conjugate approved for use in aUC.^8,15,16^ Sacituzumab govitecan, another antibody-drug conjugate consisting of a monoclonal antibody targeting trophoblast-antigen-2 linked to the active metabolite of irinotecan (SN-38), has received accelerated approval for the treatment of patients with aUC who have previously undergone platinum-based chemotherapy and PD-1/PD-L1 inhibitor treatment.^8,17^

Treatment patterns outside clinical trials are likely to exhibit variations compared with those observed in the clinical trial population. Clinical trials frequently include patients with more favorable prognoses, which can complicate the generalizability of treatment patterns to a broader and more diverse patient population.^18^ Data derived from a patient population outside a clinical trial may serve as a valuable adjunct to the knowledge accrued through clinical trials, which could significantly affect patient care and contribute to advances in therapeutic development.^19,20^ In this study, we used a nationwide, deidentified database to evaluate the treatment patterns and attrition rates in patients with aUC in oncology clinics across the US.

## Methods

This cohort study was approved by the Institutional Review Board at the University of Utah (a National Cancer Institute–Comprehensive Cancer Center), which did not require informed consent owing to the use of deidentified data, and fully complied with the US patient confidentiality regulations, including adherence to the Health Insurance Portability and Accountability Act of 1996. The study followed the Strengthening the Reporting of Observational Studies in Epidemiology (STROBE) reporting guideline.

### Study Design

We conducted a retrospective cohort study with extracted patient data from the Flatiron Health electronic health record–derived database.^19,21^ The longitudinal Flatiron Health database is composed of deidentified patient-level structured and unstructured data and curated via technology-enabled abstraction.^22,23^ During the study period, the deidentified data originated from approximately 280 oncology clinics (around 800 sites of care) across the US.^21,24^ Most patients in the database originate from community oncology settings; relative proportions of community to academic settings may vary depending on the study cohort. The electronic health record data are subjected to anonymization procedures and encompass structured data such as cancer-related diagnoses, disease staging, medication records, and abstracted information extracted from unstructured sources, notably physicians’ clinical notes.

### Patient Population

Patients were eligible to be included in the study if they had a diagnosis consistent with aUC. Patients who did not receive first-line therapy, who received treatment for 2 or more cancers, or who were participating in clinical trials in any line of therapy were excluded. In our analytic cohort, patients received treatment for aUC at the participating site from January 1, 2011, to January 31, 2023. Patients received first-line treatment from January 25, 2011, to January 31, 2023, second-line treatment from April 4, 2011, to January 31, 2023, and third-line treatment from May 23, 2011, to January 31, 2023. The treatments were categorized based on National Comprehensive Cancer Network–approved regimens into cisplatin-based regimens, carboplatin-based regimens, PD-1/PD-L1 inhibitors, single-agent nonplatinum chemotherapy, enfortumab vedotin, erdafitinib, sacituzumab govitecan, and others. Attrition was defined as the percentage of patients not progressing to the following line of therapy.

### Statistical Analysis

Treatments in each line of therapy were summarized using frequency and percentages. All analyses were performed using R, version 4.2.3 (R Project for Statistical Computing).

## Results

Of the 12 157 patients included from the database with aUC, 8660 had information on lines of therapy. After excluding patients who received treatment for 2 or more cancers or were enrolled in clinical trials, 7260 patients were included in the final analysis cohort (5364 [73.9%] men and 1894 [26.1%] women, with data missing for 2; median age at the start of first-line treatment, 73 [IQR, 66-80] years) (eFigure 1 in Supplement 1). With regard to race and ethnicity distribution, 282 participants (3.9%) were Hispanic or Latino, 84 (1.2%) were non-Hispanic Asian, 319 (4.4%) were non-Hispanic Black, 4957 (68.3%) were non-Hispanic White, 910 (12.5%) were of other race or ethnicity, and 708 (9.8%) were of unknown race or ethnicity. The focus was not on disparities but on significant attrition rates.

**Figure 1.**
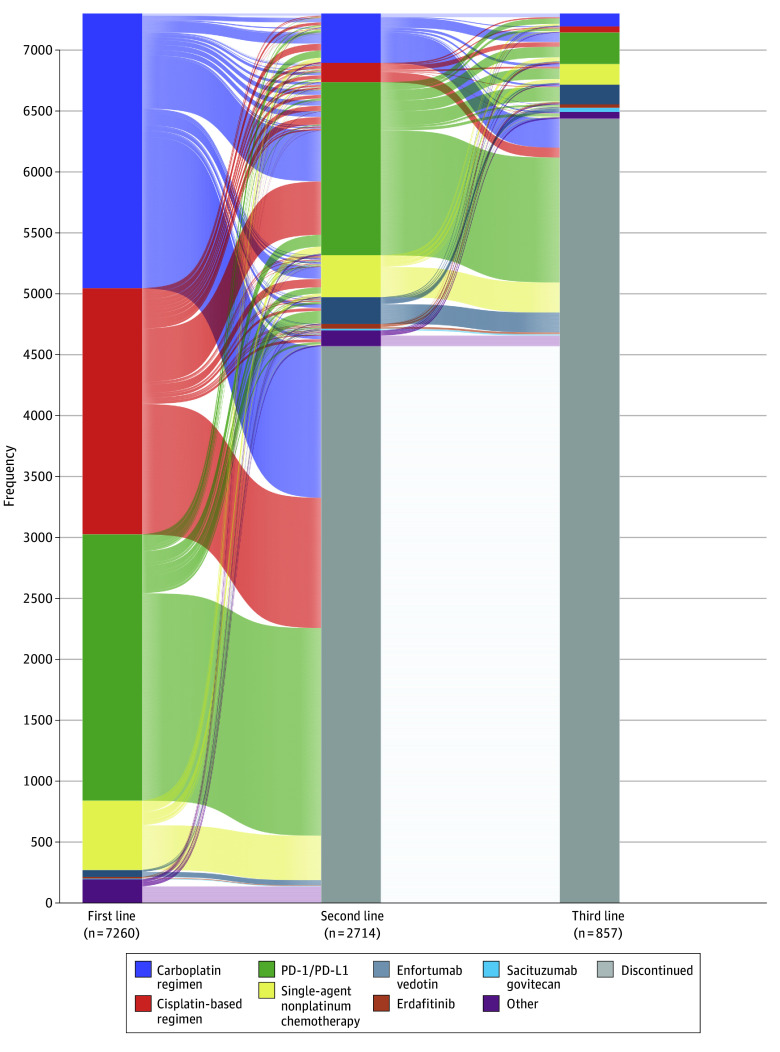
First- to Third-Line Treatment Patterns for Advanced Urothelial Cancer The Sankey diagram shows carboplatin is the most common regimen in first-line treatment, whereas programmed cell death 1 and/or programmed cell death ligand 1 (PD-1/PD-L1) is most common second-line treatment.

Of the patients in the analysis cohort, 7260 received 1 line of therapy, 2714 (37.4%) received 2 lines, and 857 (11.8%) received 3 or more lines. A breakdown of the first 6 lines of therapy is presented in the Table. Most patients who received PD-1/PD-L1 inhibitor therapy in the first line of treatment did not receive any further lines. eFigure 2 in Supplement 1 depicts the percentage of patients with aUC receiving first, second, and third lines of therapy. Figure 1 shows the treatment sequencing by lines of therapy.

**Table.** Treatment Landscape in Advanced Urothelial Cancer From First to Sixth Line

Treatment	Treatment line, No. (%) of patients (n = 7260)					
	First	Second	Third	Fourth	Fifth	Sixth
All	7260 (100)	2714 (37.4)	857 (11.8)	282 (3.9)	81 (1.1)	27 (0.4)
Carboplatin-based regimen	2241 (30.9)	403 (14.8)	106 (12.4)	28 (9.9)	7 (8.6)	2 (7.4)
Cisplatin-based regimen	2008 (27.7)	157 (5.8)	48 (5.6)	10 (3.5)	0	2 (7.4)
PD-1/PD-L1 inhibitors	2174 (29.9)	1412 (52.0)	258 (30.1)	75 (26.6)	13 (16.0)	5 (18.5)
Single-agent nonplatinum chemotherapy	565 (7.8)	342 (12.6)	169 (19.7)	47 (16.7)	23 (28.4)	7 (25.9)
Enfortumab vedotin	57 (0.8)	219 (8.1)	159 (18.6)	62 (22.0)	13 (16.0)	2 (7.4)
Erdafitinib	14 (0.2)	39 (1.4)	28 (3.3)	8 (2.8)	4 (4.9)	3 (11.1)
Sacituzumab govitecan	6 (0.1)	14 (0.5)	34 (4.0)	27 (9.6)	15 (18.5)	1 (3.7)
Other	195 (2.7)	128 (4.7)	55 (6.4)	25 (8.9)	6 (7.4)	5 (18.5)

Abbreviation: PD-1/PD-L1, programmed cell death 1 and/or programmed cell death ligand 1.

A subanalysis was conducted to assess attrition rates over time. The cohort was divided into patients who started first-line therapy between January 1, 2011, and May 17, 2016, and those who began first-line therapy between May 18, 2016, and January 31, 2023. The point of May 18, 2016, was chosen due to the approval of atezolizumab by the US Food and Drug Administration (FDA) on this date, and the approval of newer, better-tolerated therapies could potentially affect the attrition rates. In the first group (January 1, 2011, to May 17, 2016), 2166 patients received a first line of therapy; of those patients, 795 (36.7%) received a second line and 257 (11.9%) received a third line of therapy. In the second group (May 18, 2016, to January 31, 2023), 5094 received a first line of therapy; of those patients, 1919 (37.7%) received a second line and 600 (11.8%) received a third line of therapy.

### Treatment Landscape in the First-Line Setting

In the first-line setting, carboplatin was the most commonly used regimen (2241 [30.9%]), followed by PD-1/PD-L1 inhibitors (2174 [29.9%]) and cisplatin-based regimens (2008 [27.7%]). eFigure 3 in Supplement 1 displays the frequency of different regimens in the first-line setting.

**Figure 2.**
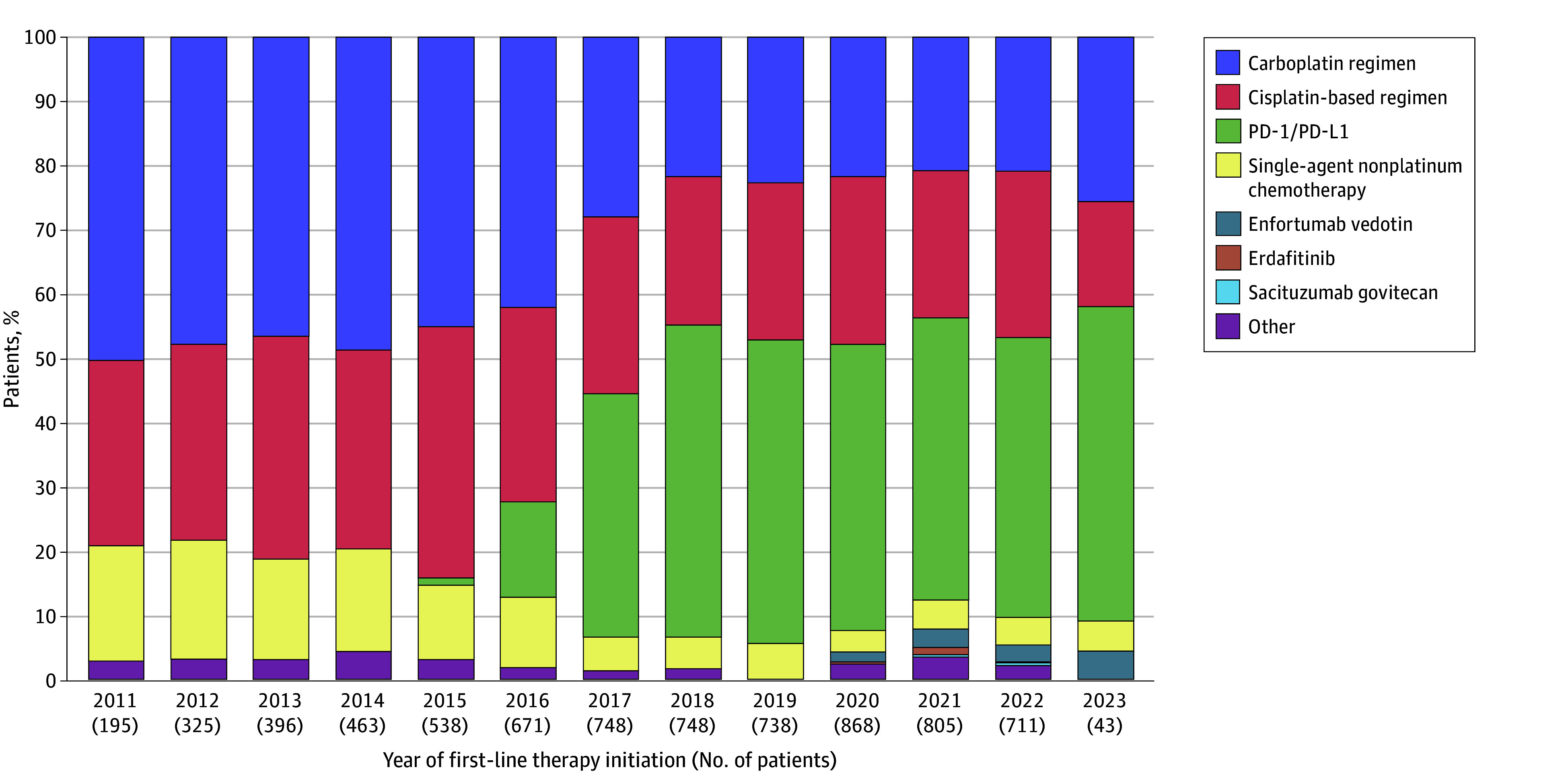
First-Line Treatment Patterns for Advanced Urothelial Cancer From 2011 to 2023 PD-1/PD-L1 indicates programmed cell death 1 and/or programmed cell death ligand 1.

Use of platinum-based chemotherapy declined starting in 2016, with a corresponding increase in PD-1/PD-L1 inhibitor therapy in the first line. These changes plateaued around 2018. Figure 2 shows the patterns of first-line treatment for aUC from 2011 to 2023.

### Treatment Landscape in the Second-Line Setting

**Figure 3.**
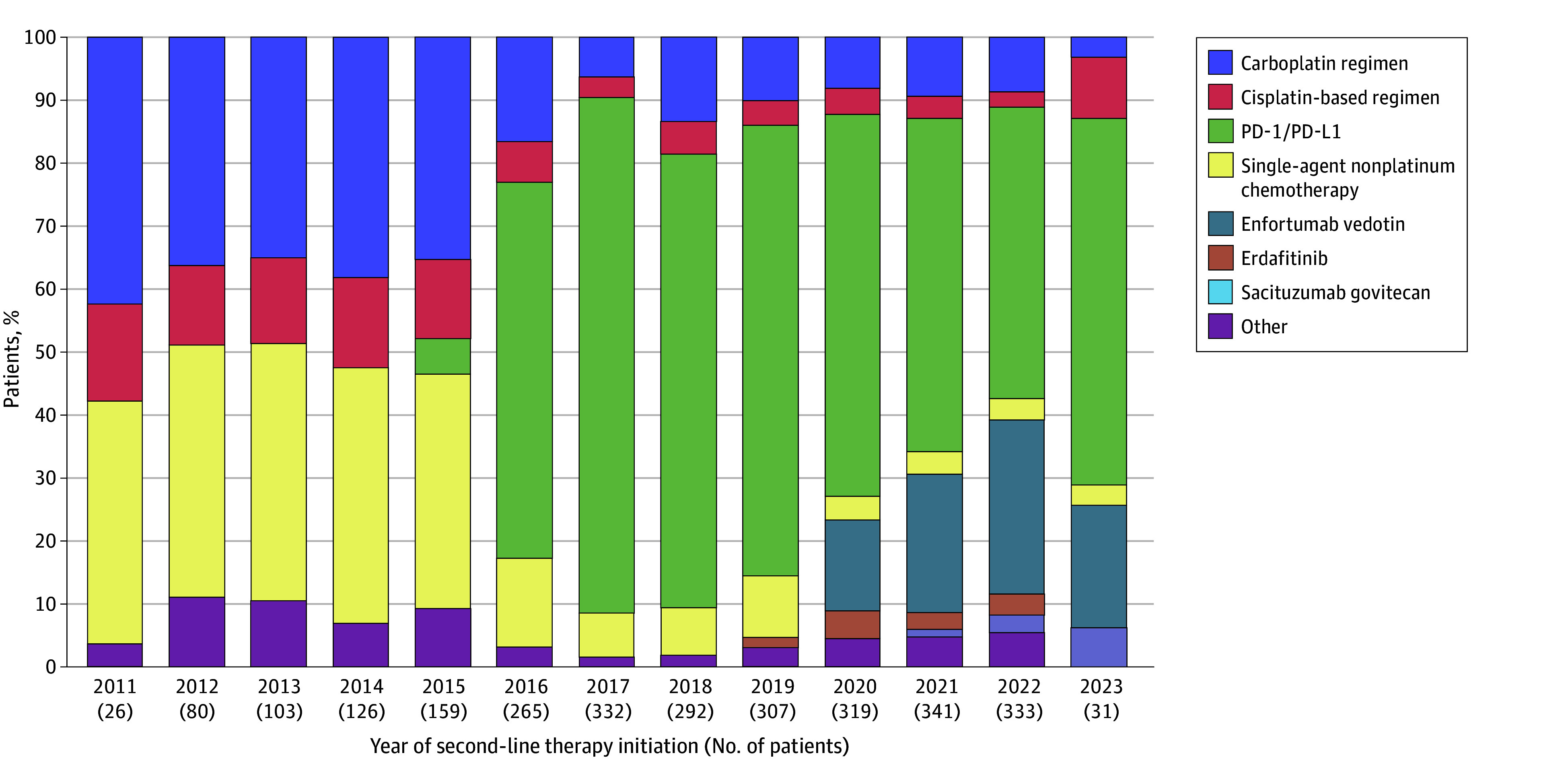
Second-Line Treatment Patterns for Advanced Urothelial Cancer From 2011 to 2023 PD-1/PD-L1 indicates programmed cell death 1 and/or programmed cell death ligand 1.

Therapy consisting of PD-1/PD-L1 inhibitors was the most commonly used in the second line (1412 [52.0%]), followed by carboplatin (403 [14.8%]) and single-agent nonplatinum chemotherapy (342 [12.6%]). eFigure 4 in Supplement 1 displays the frequency of regimens used in the second-line setting. The uptake of PD-1/PD-L1 inhibitor therapy increased starting in 2016 compared with previous years, with a corresponding decrease in the use of platinum-based chemotherapy. Since 2019, a consistent rise in the use of novel agents like enfortumab vedotin (219 [8.1%]), erdafitinib (39 [1.4%]), and sacituzumab govitecan (14 [0.5%]) occurred. Figure 3 shows the patterns of second-line treatment for aUC from 2011 to 2023.

### Treatment Landscape in the Third-Line Setting

Therapy based on PD-1/PD-L1 inhibitors represented the predominant third-line treatment regimen (258 [30.1%]), followed by single-agent nonplatinum chemotherapy (169 [19.7%]) and novel agent enfortumab vedotin (159 [18.6%]). eFigure 5 in Supplement 1 displays the frequency of use of each regimen in the third-line setting.

**Figure 4.**
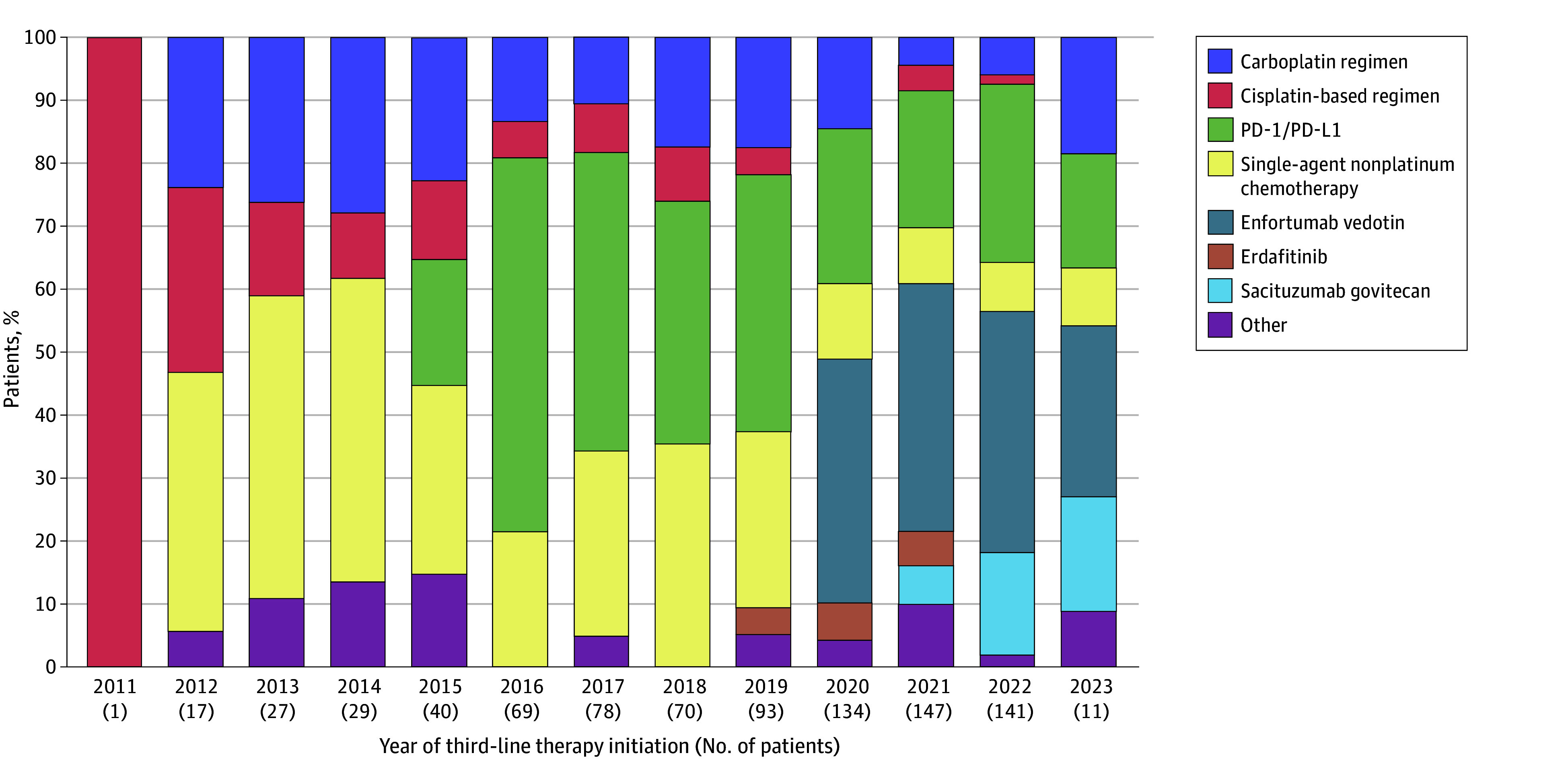
Third-Line Treatment Patterns for Advanced Urothelial Cancer From 2011 to 2023 PD-1/PD-L1 indicates programmed cell death 1 and/or programmed cell death ligand 1.

The use of platinum chemotherapy decreased with a corresponding increase in PD-1/PD-L1 inhibitor therapy from 2015. Since 2019, a consistent increase in the use of novel therapies has occurred, similar to the second-line setting, including enfortumab vedotin as noted above, erdafitinib (28 [3.3%]), and sacituzumab govitecan (34 [4.0%]). Figure 4 shows the patterns of third-line treatment for aUC from 2011 to 2023.

## Discussion

Using the Flatiron Health database, we evaluated treatment patterns and attrition rates in aUC from 2011 to 2023. Carboplatin-based regimens are the most commonly used in first-line treatment, although there has been a gradual increase in the use of PD-1/PD-L1 inhibitor therapy since 2016. Therapy with PD-1/PD-L1 inhibitors is the predominant choice in second and third lines. The adoption of novel therapies has gradually increased in the second and third lines. An alarmingly high attrition rate was observed in the cohort, with only 37.4% receiving 2 or more lines of treatment and merely 11.8% receiving 3 or more.

In first-line treatment, carboplatin emerged as the most frequently used therapeutic regimen. Notably, while cisplatin represents a current standard of care for first-line treatment for aUC among eligible patients, carboplatin serves as an acceptable alternative for patients ineligible for cisplatin-based therapy. Galsky et al^25^ conducted a meta-analysis of 4 randomized trials,^26,27,28,29^ revealing that cisplatin-based regimens have better response rates than carboplatin-containing regimens. Prior studies^19,30,31,32,33^ have indicated a notably increased use of carboplatin in first-line treatment. This can be a result of cisplatin ineligibility in most patients and concerns for cisplatin toxicity in an elderly population with comorbidities and a lack of social support.^33^

The use of PD-1/PD-L1 inhibitor therapy in the first line began increasing in 2016, with a notably substantial rise observed in 2017. In 2017, the US FDA granted accelerated approval to first-line atezolizumab for patients with aUC who were ineligible for cisplatin, based on the results of cohort 1 of the phase 2 IMvigor210 study.^8,34^ In parallel, in 2017, pembrolizumab was granted accelerated approval for the same indication based on the phase 2 KEYNOTE-052 trial results.^8,35^ In 2018, the FDA issued a safety advisory concerning the use of pembrolizumab and atezolizumab as monotherapy in first-line treatment. This advisory was prompted by preliminary analyses of data from 2 ongoing clinical trials, specifically the KEYNOTE-361 and IMvigor130 trials, which revealed decreased overall survival among patients administered pembrolizumab or atezolizumab compared with those who received cisplatin- or carboplatin-based therapeutic regimens.^12,36^ Subsequently, based on data from these trials, the indication for pembrolizumab was amended to its current form, and the manufacturers of atezolizumab voluntarily withdrew its indication in first-line treatment of aUC in 2022.^8^ Avelumab, approved in 2020 for maintenance therapy in aUC, likely also contributes to a proportion of PD-1/PD-L1 inhibitors used in our study.^8,13^

In second-line treatment, PD-1/PD-L1 inhibitor therapy was the most commonly used regimen. Between 2011 and 2015, platinum-based therapy and single-agent nonplatinum chemotherapy predominated. However, a notable shift occurred in 2016, marked by a pronounced increase in the adoption of PD-1/PD-L1 inhibitors. In 2016, atezolizumab received accelerated approval for the treatment of patients with aUC who had experienced disease progression after or during platinum-based chemotherapy, based on the outcomes of cohort 2 within the phase 2 IMvigor210 trial.^8,37^ This approval, coupled with the favorable safety profile of atezolizumab and patient perception, likely contributed to the heightened adoption of this approach.^37,38^ Following atezolizumab’s approval, nivolumab and avelumab also received accelerated approvals, while pembrolizumab was granted regular approval in 2017 for the same indication as atezolizumab.^8^ However, in 2021, the manufacturers of atezolizumab voluntarily withdrew its indication for use in aUC postplatinum therapy based on the results of the phase 3 IMvigor211 trial that failed to show a survival benefit with atezolizumab compared with single-agent chemotherapy.^8,39^ The use of erdafitinib displayed a notable upswing in 2019, concomitant with the accelerated approval of erdafitinib in the same year.^8^ Enfortumab vedotin received accelerated approval in December 2019 for the treatment of patients with aUC who had previously undergone platinum-based chemotherapy and PD-1/PD-L1 inhibitor therapy, predicated on the results of the phase 2 EV-201 trial.^8,40^ The approval corresponded to increased use, as shown in Figure 3 . In 2021, the FDA expanded the approved indication for enfortumab vedotin in aUC to encompass patients who were ineligible for cisplatin and had experienced disease progression following at least 1 line of therapy.^8^ Use of sacituzumab govitecan increased in 2021, in alignment with its accelerated approval in the same year.^8^ Notably, we also noticed a decline in the use of erdafitinib with the increasing use of enfortumab vedotin and sacituzumab govitecan. The observations in third-line treatment mirrored those in the second line, wherein PD-1/PD-L1 inhibitors remained the predominant choice, and a discernible rise in the use of novel therapeutic agents commenced in 2019.

One of the most startling findings in our study pertained to the elevated attrition rates, wherein merely 37.4% of patients received a minimum of 2 lines of therapy, and 11.8% received at least 3 lines. Our subanalysis found similar attrition rates in patients starting first-line treatment for aUC from January 1, 2011, to May 17, 2016, and those starting first-line treatment from May 18, 2016, to January 31, 2023. These high attrition rates align with findings from prior studies on aUC.^18,19,30,33^ Multiple factors may contribute to these high attrition rates, including socioeconomic variables, difficulties in accessing affordable health care, and the use of treatment regimens characterized by poor tolerability and modest efficacy.^33^ A promising strategy for optimizing patient treatment is the implementation of front-line therapeutic regimens known for their effectiveness and favorable tolerability profiles. The phase 3 EV-302–KEYNOTE-A39 trial^41^ exemplifies such an approach, where the combination of enfortumab vedotin and pembrolizumab was compared with gemcitabine plus platinum chemotherapy for previously untreated locally advanced or metastatic urothelial carcinoma. The results of the trial showed a significant improvement in progression-free survival (PFS) (median PFS, 12.5 [95% CI, 10.4-16.6] vs 6.3 [95% CI, 6.2-6.5] months; hazard ratio [HR], 0.45 [95% CI, 0.38-0.54]) and overall survival (OS) (median OS, 31.5 [95% CI, 25.4 to not reached] vs 16.1 [95% CI, 13.9-18.3] months; HR, 0.47 [95% CI, 0.38-0.58]) in favor of the enfortumab vedotin plus pembrolizumab arm. Moreover, fewer grade 3 treatment-related adverse events were observed in the enfortumab vedotin plus pembrolizumab arm.^41^ These compelling results are anticipated to position the combination of enfortumab vedotin and pembrolizumab as the new standard of care in first-line treatment of aUC at the potential expense of platinum-based chemotherapy. Nevertheless, it remains uncertain whether enfortumab vedotin with pembrolizumab is the optimal choice for patients who have undergone prior anti–PD-1 therapy in the adjuvant setting. Makrakis et al^42^ conducted a retrospective study revealing that patients receiving a second immune checkpoint inhibitor (ICI) after progression during initial ICI treatment demonstrated an overall response rate of 13%. This finding implies that a limited subset of patients rechallenged with an ICI may experience clinical benefits, but whether enfortumab vedotin with pembrolizumab will provide additional benefit compared with single-agent enfortumab needs to be explored. Clinical trials are essential to address this crucial question.^42^ For patients for whom enfortumab vedotin with pembrolizumab is not a viable option, an alternative choice is nivolumab in combination with gemcitabine and cisplatin. CheckMate 901,^43^ a phase 3 trial, compared nivolumab plus gemcitabine and cisplatin with gemcitabine and cisplatin alone. Adding nivolumab produced a statistically significant increase in the median PFS (7.9 [95% CI, 7.6-9.5] vs 7.6 [95% CI, 6.1-7.8] months; HR, 0.72 [95% CI, 0.59-0.88]) and median OS (21.7 [95% CI, 18.6-26.4] vs 18.9 [95% CI, 14.7-22.4] months; HR, 0.78 [95% CI, 0.63-0.96]). In the nivolumab group, 62% experienced grade 3 or higher adverse effects, compared with 52% in the gemcitabine plus cisplatin group.^43^ The ongoing NILE trial^44^ is evaluating durvalumab with or without tremelimumab in combination with platinum-based chemotherapy as first-line treatment for locally advanced unresectable and metastatic urothelial cancer. Future trials incorporating predictive biomarkers for guiding treatment selection could potentially refine treatment choices in patients with aUC.

Even with these advances, attrition rates may continue to be high, and a comprehensive, patient-centric approach needs to be made to make a difference. This will not only entail bringing effective and well-tolerated therapies at reasonable cost in earlier disease settings but also improving social support and use of novel ways through information and technology to deliver patient care near their residence.^33^

### Strengths and Limitations

As our study data were obtained from a population outside a clinical trial, our results can be generalized to a broad and diverse patient population. Limitations of our study include its retrospective nature and only US-based population. The absence of data regarding the baseline characteristics of patients, including patient preference and comorbidities, is notable, rendering us unable to discern the specific factors contributing to cisplatin ineligibility in first-line treatment. Other limitations of our study include the lack of data on clinical outcomes, a lack of randomization, potential selection bias, and various unmeasured confounders that may influence treatment selection.

## Conclusions

In this cohort study, carboplatin emerged as the prevailing regimen in first-line treatment, while PD-1/PD-L1 inhibitors predominated in second- and third-line treatments. We observed a growing adoption of novel therapeutic agents following their regulatory approvals. Notably, the study also underscored an exceptionally high attrition rate among patients. Addressing this issue necessitates developing front-line treatment regimens that are both better tolerated and more effective and could potentially improve patient outcomes.

## Notes

Supplement 1. **eFigure 1.** STROBE Flow Diagram **eFigure 2.** Illustration of Patients With Advanced Urothelial Carcinoma Receiving First, Second, and Third Lines of Therapy **eFigure 3.** Frequency of Use of Different Regimens in the First-Line Setting **eFigure 4.** Frequency of Use of Different Regimens in the Second-Line Setting **eFigure 5.** Frequency of Use of Different Regimens in the Third-Line Setting

Supplement 2.
Data Sharing Statement

## References

[1] Siegel RL, Miller KD, Wagle NS, Jemal A. Cancer statistics, 2023. CA Cancer J Clin. 2023;73(1):17-48. doi:10.3322/caac.21763 36633525

[2] National Institute of Health, National Cancer Institute, Surveillance, Epidemiology, and End Results Program. Cancer stat facts: bladder cancer. 2023. Accessed October 10, 2023. https://seer.cancer.gov/statfacts/html/urinb.html

[3] Humphrey PA, Moch H, Cubilla AL, Ulbright TM, Reuter VE. The 2016 WHO classification of tumours of the urinary system and male genital organs–part B: prostate and bladder tumours. Eur Urol. 2016;70(1):106-119. doi:10.1016/j.eururo.2016.02.028 26996659

[4] Moore MJ, Iscoe N, Tannock IF. A phase II study of methotrexate, vinblastine, doxorubicin and cisplatin plus recombinant human granulocyte-macrophage colony stimulating factors in patients with advanced transitional cell carcinoma. J Urol. 1993;150(4):1131-1134. doi:10.1016/S0022-5347(17)35706-3 8371371

[5] Sternberg CN, de Mulder P, Schornagel JH, ; EORTC Genito-Urinary Cancer Group. Seven year update of an EORTC phase III trial of high-dose intensity M-VAC chemotherapy and G-CSF versus classic M-VAC in advanced urothelial tract tumours. Eur J Cancer. 2006;42(1):50-54. doi:10.1016/j.ejca.2005.08.032 16330205

[6] von der Maase H, Hansen SW, Roberts JT, . Gemcitabine and cisplatin versus methotrexate, vinblastine, doxorubicin, and cisplatin in advanced or metastatic bladder cancer: results of a large, randomized, multinational, multicenter, phase III study. J Clin Oncol. 2023;41(23):3881-3890. doi:10.1200/JCO.22.02763 37549482

[7] De Santis M, Bellmunt J, Mead G, . Randomized phase II/III trial assessing gemcitabine/carboplatin and methotrexate/carboplatin/vinblastine in patients with advanced urothelial cancer “unfit” for cisplatin-based chemotherapy: phase II—results of EORTC study 30986. J Clin Oncol. 2009;27(33):5634-5639. doi:10.1200/JCO.2008.21.4924 19786668 PMC2792956

[8] United States Food and Drug Administration. Oncology (cancer)/hematologic malignancies approval notifications. Accessed November 13, 2023. https://www.fda.gov/drugs/resources-information-approved-drugs/oncology-cancer-hematologic-malignancies-approval-notifications

[9] Bellmunt J, de Wit R, Vaughn DJ, ; KEYNOTE-045 Investigators. Pembrolizumab as second-line therapy for advanced urothelial carcinoma. N Engl J Med. 2017;376(11):1015-1026. doi:10.1056/NEJMoa1613683 28212060 PMC5635424

[10] Patel MR, Ellerton J, Infante JR, . Avelumab in metastatic urothelial carcinoma after platinum failure (JAVELIN Solid Tumor): pooled results from two expansion cohorts of an open-label, phase 1 trial. Lancet Oncol. 2018;19(1):51-64. doi:10.1016/S1470-2045(17)30900-2 29217288 PMC7984727

[11] Sharma P, Retz M, Siefker-Radtke A, . Nivolumab in metastatic urothelial carcinoma after platinum therapy (CheckMate 275): a multicentre, single-arm, phase 2 trial. Lancet Oncol. 2017;18(3):312-322. doi:10.1016/S1470-2045(17)30065-7 28131785

[12] Powles T, Csőszi T, Özgüroğlu M, ; KEYNOTE-361 Investigators. Pembrolizumab alone or combined with chemotherapy versus chemotherapy as first-line therapy for advanced urothelial carcinoma (KEYNOTE-361): a randomised, open-label, phase 3 trial. Lancet Oncol. 2021;22(7):931-945. doi:10.1016/S1470-2045(21)00152-2 34051178

[13] Powles T, Park SH, Voog E, . Avelumab maintenance therapy for advanced or metastatic urothelial carcinoma. N Engl J Med. 2020;383(13):1218-1230. doi:10.1056/NEJMoa2002788 32945632

[14] Loriot Y, Necchi A, Park SH, ; BLC2001 Study Group. Erdafitinib in locally advanced or metastatic urothelial carcinoma. N Engl J Med. 2019;381(4):338-348. doi:10.1056/NEJMoa1817323 31340094

[15] Hoimes CJ, Flaig TW, Milowsky MI, . Enfortumab vedotin plus pembrolizumab in previously untreated advanced urothelial cancer. J Clin Oncol. 2023;41(1):22-31. doi:10.1200/JCO.22.01643 36041086 PMC10476837

[16] Powles T, Rosenberg JE, Sonpavde GP, . Enfortumab vedotin in previously treated advanced urothelial carcinoma. N Engl J Med. 2021;384(12):1125-1135. doi:10.1056/NEJMoa2035807 33577729 PMC8450892

[17] Tagawa ST, Balar AV, Petrylak DP, . TROPHY-U-01: a phase II open-label study of sacituzumab govitecan in patients with metastatic urothelial carcinoma progressing after platinum-based chemotherapy and checkpoint inhibitors. J Clin Oncol. 2021;39(22):2474-2485. doi:10.1200/JCO.20.03489 33929895 PMC8315301

[18] Richters A, Mehra N, Meijer RP, . Utilization of systemic treatment for metastatic bladder cancer in everyday practice: results of a nation-wide population-based cohort study. Cancer Treat Res Commun. 2020;25:100266. doi:10.1016/j.ctarc.2020.100266 33316557

[19] Simeone JC, Nordstrom BL, Patel K, Mann H, Klein AB, Horne L. Treatment patterns and overall survival in metastatic urothelial carcinoma in a real-world, US setting. Cancer Epidemiol. 2019;60:121-127. doi:10.1016/j.canep.2019.03.013 30953972

[20] Sherman RE, Anderson SA, Dal Pan GJ, . Real-world evidence—what is it and what can it tell us? N Engl J Med. 2016;375(23):2293-2297. doi:10.1056/NEJMsb1609216 27959688

[21] Flatiron Health. Accessed October 18, 2023. https://flatiron.com/

[22] Ma X, Long L, Moon S, Adamson BJS, Baxi SS. Comparison of population characteristics in real-world clinical oncology databases in the US: Flatiron Health, SEER, and NPCR. medRxiv. Preprint posted online January 25, 2023. doi:10.1101/2020.03.16.20037143

[23] Birnbaum B, Nussbaum N, Seidl-Rathkopf K, . Model-assisted cohort selection with bias analysis for generating large-scale cohorts from the EHR for oncology research. arXiv. Preprint posted online January 2020. doi:10.48550/arXiv.2001.09765

[24] Graf RP, Fisher V, Mateo J, . Predictive genomic biomarkers of hormonal therapy versus chemotherapy benefit in metastatic castration-resistant prostate cancer. Eur Urol. 2022;81(1):37-47. doi:10.1016/j.eururo.2021.09.030 34716049

[25] Galsky MD, Chen GJ, Oh WK, . Comparative effectiveness of cisplatin-based and carboplatin-based chemotherapy for treatment of advanced urothelial carcinoma. Ann Oncol. 2012;23(2):406-410. doi:10.1093/annonc/mdr156 21543626

[26] Petrioli R, Frediani B, Manganelli A, . Comparison between a cisplatin-containing regimen and a carboplatin-containing regimen for recurrent or metastatic bladder cancer patients: a randomized phase II study. Cancer. 1996;77(2):344-351. doi:10.1002/(SICI)1097-0142(19960115)77:2<344::AID-CNCR18>3.0.CO;2-1 8625244

[27] Bellmunt J, Ribas A, Eres N, . Carboplatin-based versus cisplatin-based chemotherapy in the treatment of surgically incurable advanced bladder carcinoma. Cancer. 1997;80(10):1966-1972. doi:10.1002/(SICI)1097-0142(19971115)80:10<1966::AID-CNCR14>3.0.CO;2-W 9366300

[28] Dogliotti L, Cartenì G, Siena S, . Gemcitabine plus cisplatin versus gemcitabine plus carboplatin as first-line chemotherapy in advanced transitional cell carcinoma of the urothelium: results of a randomized phase 2 trial. Eur Urol. 2007;52(1):134-141. doi:10.1016/j.eururo.2006.12.029 17207911

[29] Dreicer R, Manola J, Roth BJ, . Phase III trial of methotrexate, vinblastine, doxorubicin, and cisplatin versus carboplatin and paclitaxel in patients with advanced carcinoma of the urothelium. Cancer. 2004;100(8):1639-1645. doi:10.1002/cncr.20123 15073851

[30] Galsky MD, Pal SK, Lin SW, . Real-world effectiveness of chemotherapy in elderly patients with metastatic bladder cancer in the United States. Bladder Cancer. 2018;4(2):227-238. doi:10.3233/BLC-170149 29732393 PMC5929305

[31] Aly A, Johnson C, Yang S, Botteman MF, Rao S, Hussain A. Overall survival, costs, and healthcare resource use by line of therapy in Medicare patients with newly diagnosed metastatic urothelial carcinoma. J Med Econ. 2019;22(7):662-670. doi:10.1080/13696998.2019.1591424 30836812 PMC7384456

[32] Flannery K, Boyd M, Black-Shinn J, Robert N, Kamat AM. Outcomes in patients with metastatic bladder cancer in the USA: a retrospective electronic medical record study. Future Oncol. 2019;15(12):1323-1334. doi:10.2217/fon-2018-0654 30942088

[33] Swami U, Grivas P, Pal SK, Agarwal N. Utilization of systemic therapy for treatment of advanced urothelial carcinoma: lessons from real world experience. Cancer Treat Res Commun. 2021;27:100325. doi:10.1016/j.ctarc.2021.100325 33549986

[34] Balar AV, Galsky MD, Rosenberg JE, ; IMvigor210 Study Group. Atezolizumab as first-line treatment in cisplatin-ineligible patients with locally advanced and metastatic urothelial carcinoma: a single-arm, multicentre, phase 2 trial. Lancet. 2017;389(10064):67-76. doi:10.1016/S0140-6736(16)32455-2 27939400 PMC5568632

[35] Balar AV, Castellano D, O’Donnell PH, . First-line pembrolizumab in cisplatin-ineligible patients with locally advanced and unresectable or metastatic urothelial cancer (KEYNOTE-052): a multicentre, single-arm, phase 2 study. Lancet Oncol. 2017;18(11):1483-1492. doi:10.1016/S1470-2045(17)30616-2 28967485

[36] Galsky MD, Arija JAA, Bamias A, ; IMvigor130 Study Group. Atezolizumab with or without chemotherapy in metastatic urothelial cancer (IMvigor130): a multicentre, randomised, placebo-controlled phase 3 trial. Lancet. 2020;395(10236):1547-1557. doi:10.1016/S0140-6736(20)30230-0 32416780

[37] Rosenberg JE, Hoffman-Censits J, Powles T, . Atezolizumab in patients with locally advanced and metastatic urothelial carcinoma who have progressed following treatment with platinum-based chemotherapy: a single-arm, multicentre, phase 2 trial. Lancet. 2016;387(10031):1909-1920. doi:10.1016/S0140-6736(16)00561-4 26952546 PMC5480242

[38] Patell R, Einstein D, Miller E, Dodge L, Halleck J, Buss M. Patient perceptions of treatment benefit and toxicity in advanced cancer: a prospective cross-sectional study. JCO Oncol Pract. 2021;17(2):e119-e129. doi:10.1200/OP.20.00517 33444075

[39] van der Heijden MS, Loriot Y, Durán I, . Atezolizumab versus chemotherapy in patients with platinum-treated locally advanced or metastatic urothelial carcinoma: a long-term overall survival and safety update from the phase 3 IMvigor211 clinical trial. Eur Urol. 2021;80(1):7-11. doi:10.1016/j.eururo.2021.03.024 33902955

[40] Rosenberg JE, O’Donnell PH, Balar AV, . Pivotal trial of enfortumab vedotin in urothelial carcinoma after platinum and anti–programmed death 1/programmed death ligand 1 therapy. J Clin Oncol. 2019;37(29):2592-2600. doi:10.1200/JCO.19.01140 31356140 PMC6784850

[41] Powles TB, Perez Valderrama B, Gupta S, . LBA6 EV-302/KEYNOTE-A39: open-label, randomized phase III study of enfortumab vedotin in combination with pembrolizumab (EV+P) vs chemotherapy (Chemo) in previously untreated locally advanced metastatic urothelial carcinoma (la/mUC). Ann Oncol. 2023;34(suppl 2):S1340. doi:10.1016/j.annonc.2023.10.106

[42] Makrakis D, Bakaloudi DR, Talukder R, . Treatment rechallenge with immune checkpoint inhibitors in advanced urothelial carcinoma. Clin Genitourin Cancer. 2023;21(2):286-294. doi:10.1016/j.clgc.2022.11.003 36481176

[43] van der Heijden MS, Sonpavde G, Powles T, ; CheckMate 901 Trial Investigators. Nivolumab plus gemcitabine-cisplatin in advanced urothelial carcinoma. N Engl J Med. 2023;389(19):1778-1789. doi:10.1056/NEJMoa2309863 37870949 PMC12314471

[44] Study of durvalumab given with chemotherapy, durvalumab in combination with tremelimumab given with chemotherapy, or chemotherapy in patients with unresectable urothelial cancer (NILE). ClinicalTrials.gov identifier: NCT03682068. Updated February 12, 2024. Accessed February 15, 2024. https://classic.clinicaltrials.gov/ct2/show/NCT03682068

