# Supplementary material for: Treatment Patterns and Attrition With Lines of Therapy for Advanced Urothelial Carcinoma in the US
Source: JAMA Netw Open. 2024 May 2;7(5):e249417. doi: 10.1001/jamanetworkopen.2024.9417 (PMC11066705; doi:10.1001/jamanetworkopen.2024.9417)
Supplement: Supplement 1. — eFigure 1. STROBE Flow Diagram eFigure 2. Illustration of Patients With Advanced Urothelial Carcinoma Receiving First, Second, and Third Lines of Therapy eFigure 3. Frequency of Use of Different Regimens in the First-Line Setting eFigure 4. Frequency of Use of Different Regimens in the Second-Line Setting eFigure 5. Frequency of Use of Different Regimens in the Third-Line Setting [file jamanetwopen-e249417-s001.pdf]

Supplementary Online Content

Mathew Thomas V, Jo Y, Tripathi N, et al. Treatment patterns and attrition with lines of therapy for advanced urothelial carcinoma in the US. *JAMA Netw Open*. 2024;7(5):e249417. doi:10.1001/jamanetworkopen.2024.9417

eFigure 1. STROBE Flow Diagram
eFigure 2. Illustration of Patients With Advanced Urothelial Carcinoma Receiving First, Second, and Third Lines of Therapy
eFigure 3. Frequency of Use of Different Regimens in the First-Line Setting
eFigure 4. Frequency of Use of Different Regimens in the Second-Line Setting
eFigure 5. Frequency of Use of Different Regimens in the Third-Line Setting

This supplementary material has been provided by the authors to give readers additional information about their work.

© 2024 Mathew Thomas V et al. *JAMA Network Open*.

eFigure 1. STROBE Flow Diagram

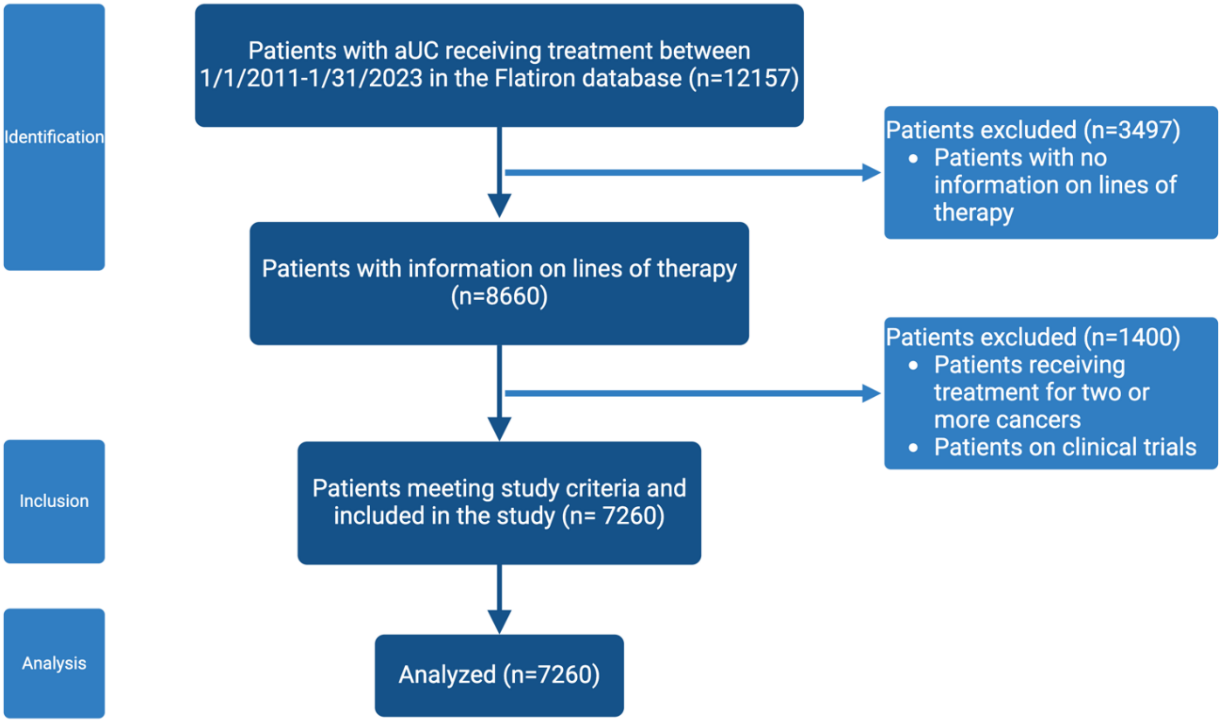

© 2024 Mathew Thomas V et al. *JAMA Network Open*.

eFigure 2. Illustration of Patients With Advanced Urothelial Carcinoma Receiving First, Second, and Third Lines of Therapy

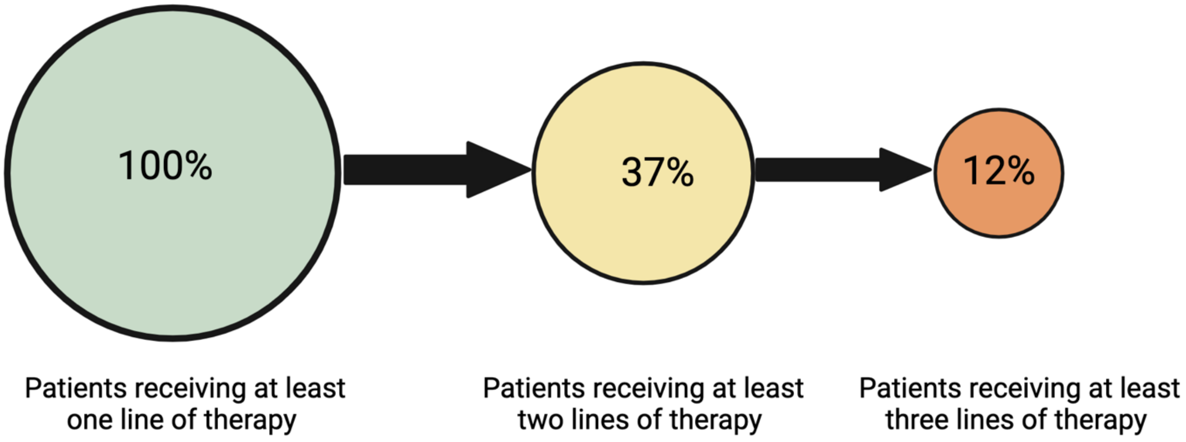

© 2024 Mathew Thomas V et al. *JAMA Network Open*.

eFigure 3. Frequency of Use of Different Regimens in the First-Line Setting

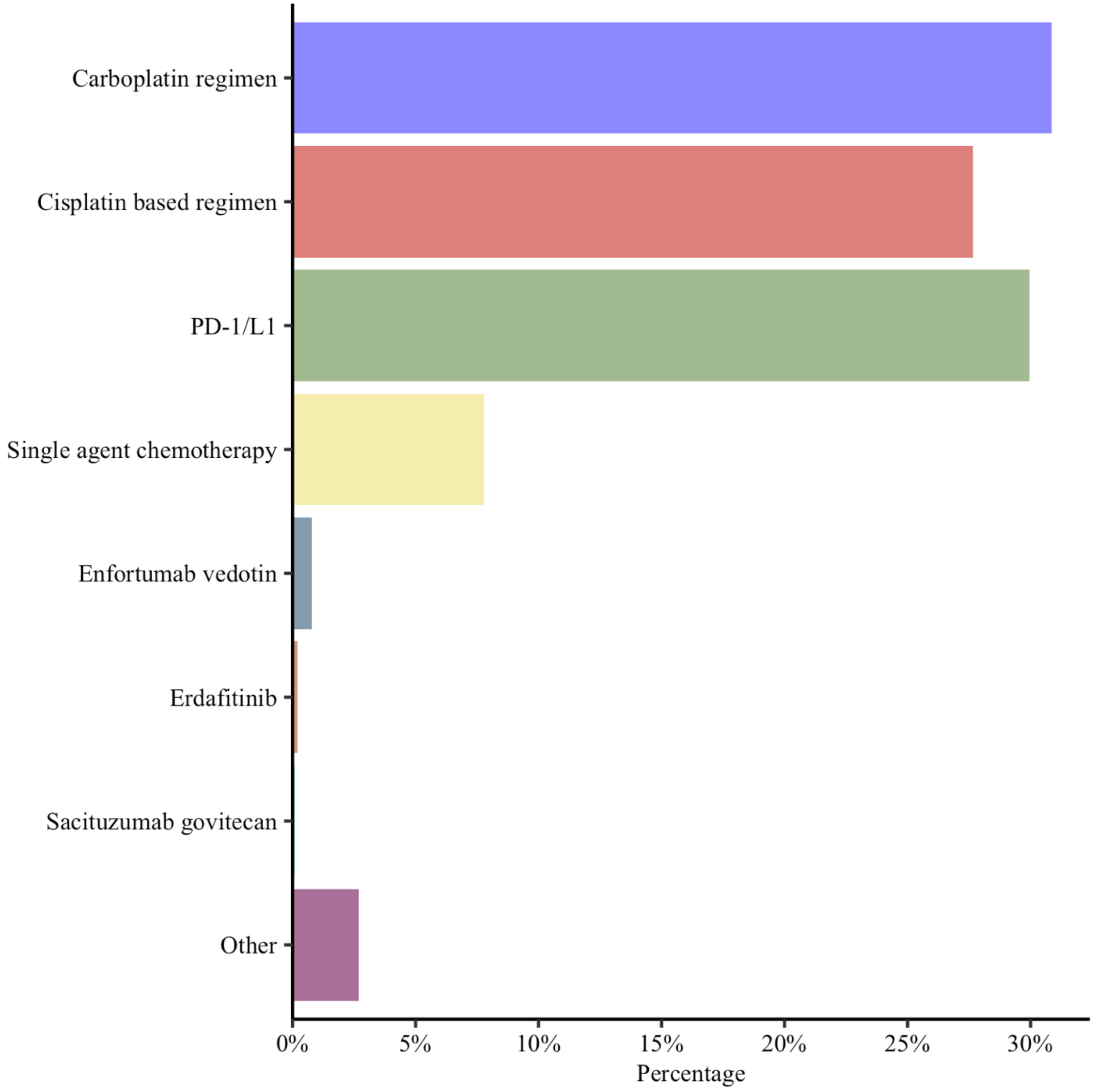

© 2024 Mathew Thomas V et al. *JAMA Network Open*.

eFigure 4. Frequency of Use of Different Regimens in the Second-Line Setting

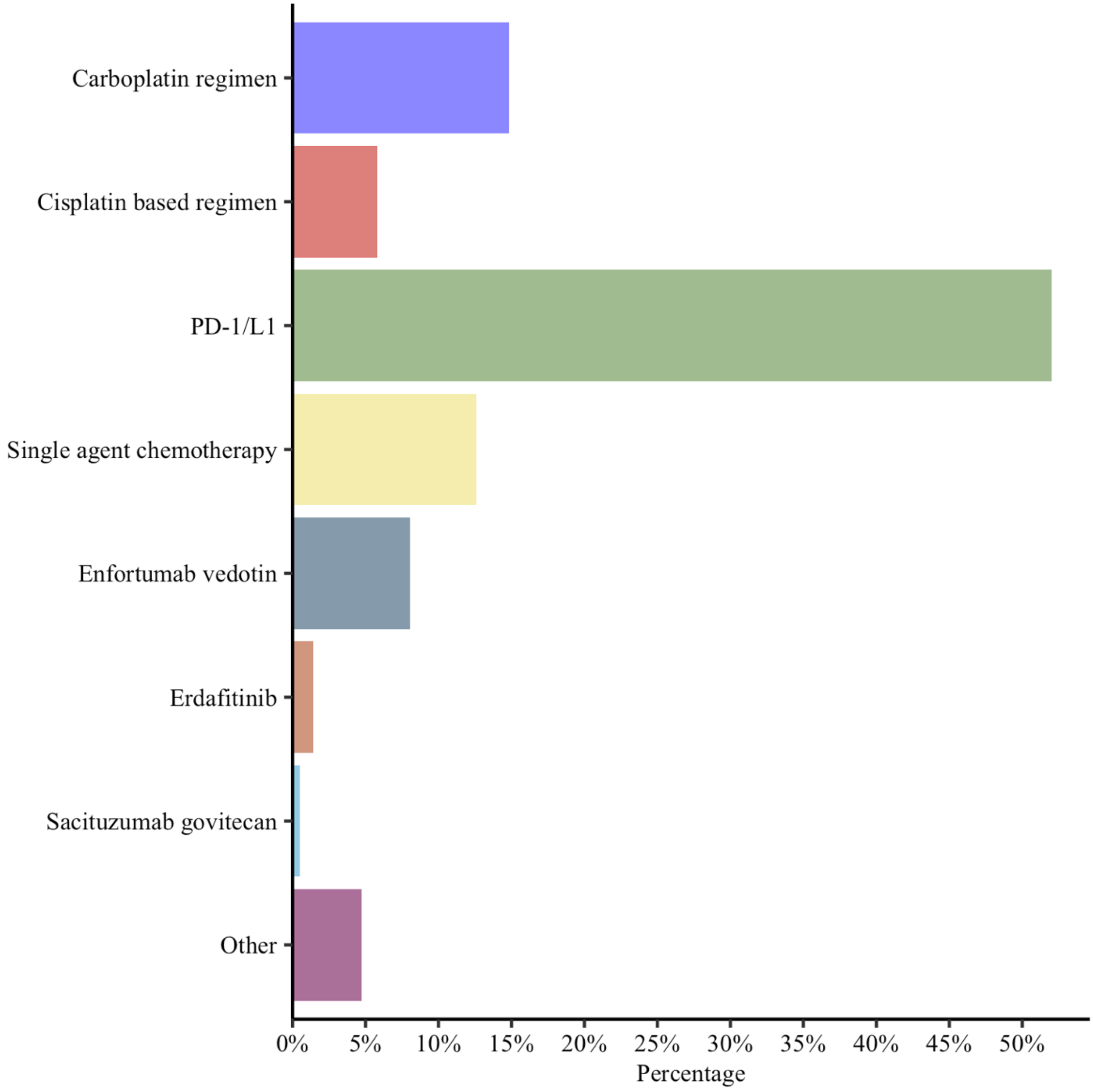

© 2024 Mathew Thomas V et al. *JAMA Network Open*.

eFigure 5. Frequency of Use of Different Regimens in the Third-Line Setting

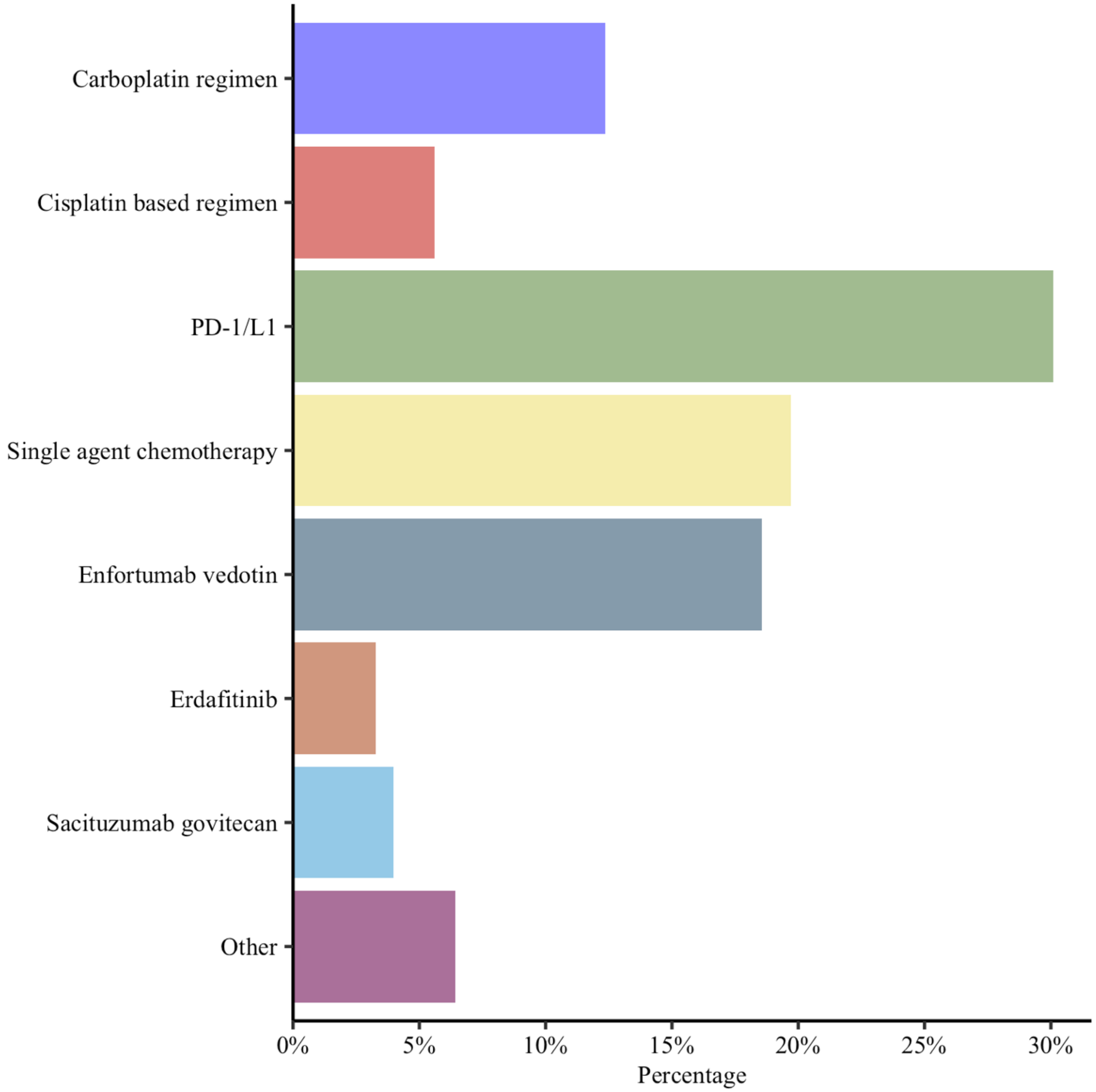

© 2024 Mathew Thomas V et al. *JAMA Network Open*.
