# Supplementary material for: Treatment Patterns and Attrition With Lines of Therapy for Advanced Urothelial Carcinoma in the US
Source: JAMA Netw Open. 2024 May 2;7(5):e249417. doi: 10.1001/jamanetworkopen.2024.9417 (PMC11066705; doi:10.1001/jamanetworkopen.2024.9417)
Supplement: Supplement 2. — Data Sharing Statement [file jamanetwopen-e249417-s002.pdf]

Data Sharing Statement

Mathew Thomas. Treatment Patterns and Attrition With Lines of Therapy for Advanced Urothelial Carcinoma in the US. *JAMA Netw Open*. Published May 02, 2024.
doi:10.1001/jamanetworkopen.2024.9417

Data
Data available: Yes
Data types: Deidentified patient data
How to access data: The research data supporting this study was provided by Flatiron Health, Inc. This deidentified data set can be accessed upon request and is governed by a licensing agreement with Flatiron Health. Researchers interested in using these data should reach out to DataAccess@flatiron.com to discuss the terms of use.
When available: With publication

Supporting Documents
Document types: None
